# Diagnosis of invasive candidiasis in the ICU

**DOI:** 10.1186/2110-5820-1-37

**Published:** 2011-09-01

**Authors:** Philippe Eggimann, Jacques Bille, Oscar Marchetti

**Affiliations:** 1 Adult Critical Care Medicine and Burn Centre, Centre Hospitalier Universitaire Vaudois (CHUV) -- BH 08-619, Bugnon 46 CH-1011 Lausanne, Switzerland; 2 Institute of Microbiology, Centre Hospitalier Universitaire Vaudois (CHUV), Lausanne, Switzerland; 3 Infectious Diseases Service, Centre Hospitalier Universitaire Vaudois (CHUV), and University of Lausanne (UNIL), Lausanne, Switzerland

## Abstract

Invasive candidiasis ranges from 5 to 10 cases per 1,000 ICU admissions and represents 5% to 10% of all ICU-acquired infections, with an overall mortality comparable to that of severe sepsis/septic shock. A large majority of them are due to *Candida albicans*, but the proportion of strains with decreased sensitivity or resistance to fluconazole is increasingly reported. A high proportion of ICU patients become colonized, but only 5% to 30% of them develop an invasive infection. Progressive colonization and major abdominal surgery are common risk factors, but invasive candidiasis is difficult to predict and early diagnosis remains a major challenge. Indeed, blood cultures are positive in a minority of cases and often late in the course of infection. New nonculture-based laboratory techniques may contribute to early diagnosis and management of invasive candidiasis. Both serologic (mannan, antimannan, and betaglucan) and molecular (Candida-specific PCR in blood and serum) have been applied as serial screening procedures in high-risk patients. However, although reasonably sensitive and specific, these techniques are largely investigational and their clinical usefulness remains to be established. Identification of patients susceptible to benefit from empirical antifungal treatment remains challenging, but it is mandatory to avoid antifungal overuse in critically ill patients. Growing evidence suggests that monitoring the dynamic of *Candida *colonization in surgical patients and prediction rules based on combined risk factors may be used to identify ICU patients at high risk of invasive candidiasis susceptible to benefit from prophylaxis or preemptive antifungal treatment.

## Epidemiology of invasive candidiasis

Whereas in the past, opportunistic mycoses, such as *Candida *and *Aspergillus*, typically occurred in immunocompromised hosts, these complications are increasingly observed in nonimmunocompromised surgical and critically ill adult patients [1,2]. These trends were confirmed by a recent large international prevalence survey in ICUs, which reported infections due to *Candida *and *Aspergillus *in 17% and 1.4% patients, respectively [3].

### Incidence of candidemia

A large epidemiological survey in the United States reported a threefold increase of fungal sepsis during the period 1979-2000, and candidemia was reported to be the third most common cause of nosocomial bloodstream infection (BSI) in critically ill adult patients, representing 11% of all BSI [4,5]. The incidence of candidemia in U.S. hospitals during 2000-2005 increased from 3.65 to 5.56 episodes per 100,000 population [6]. Incidences are usually tenfold higher in the ICUs than in other wards: 3 to 15 episodes per 10,000 ICU patients-days or 2 to 10 cases per 1,000 ICU admissions are reported, with highest rates among surgical patients [1,7].

Data from Europe have shown that the incidence of candidemia may be lower, with proportions ranging from 2-3% of bloodstream isolates [2,8]. A recent national surveillance, including 2,820 cases of fungemia in Denmark during the period 2004-2009, reported an increasing incidence from 7.7 to 8.6 per 100,000 [9]. Despite important regional differences, these data show that *Candida *is among the top ten bloodstream pathogens and suggest an increasing incidence of candidemia during the past 5 to 10 years.

### Distribution of species

A large geographical variation of the proportions of the different *Candida *species has been reported (Table 1) [2,7-16]. In North and South America, non-*albicans Candida *species account for more than half of the bloodstream isolates: *C. glabrata *and *C. parapsilosis *are the predominant non-*albicans *species, respectively. Whereas in Europe, *C. albicans *remains the most frequent species, epidemiological trends suggest that non-*albicans Candida *species, in particular *C. glabrata*, are emerging. In addition to differences in the fungal ecology of the different continents, the large use of azoles antifungal agents may have contributed to this progressive shift of the epidemiology of candidemia.

**Table 1:** Distribution of Candida species in epidemiological surveys during the past decades

Author	Period of observation	*Study*	Region	No. of strains	*Candida* *albicans*	*Candida* *tropicalis*	*Candida* *parapsilosis*	*Candida* *glabrata*	*Candida krusei*	Other *Candida*
Pfaller et al. [10]	2008-2009	SENTRY	Worldwide	2'085	48%	11%	17%	18%	2%	4%
			Europe	750	55%	7%	14%	16%	3%	4%
			North America	936	43%	11%	17%	24%	2%	4%
			Latin America	348	44%	17%	26%	5%	1%	5%
			Asia	51	57%	12%	14%	14%	2%	2%
Marra et al. [11]	2007-2010	SCOPE	Brazil	137	34%	15%	24%	10%	2%	17%
Arendrup et al. [9]	2004-2007		Denmark	2901	57%	5%	4%	21%	4%	9%
										
Horn et al. [12]	2004-2008	PATH	North America	2019	46%	8%	16%	26%	3%	1%
Leroy et al. [7]	2005-2006	AmarCand	France ICU	305	57%	5%	8%	17%	5%	8%
Talarmin et al. [13]	2004		France West	193	55%	5%	13%	19%	4%	4%
Bougnoux et al. [14]	2001-2002		Paris ICU	57	54%	9%	14%	17%	4%	2%
Marchetti et al. [2]	1991-2000	FUNGINOS	Switzerland	1137	64%	9%	1%	15%	2%	9%
Sandven et al. [15]	1991-2003		Norway Nationwide	1393	70%	7%	6%	13%	1%	3%
Pfaller et al. [16]	1997-2005	ARTEMIS	Mondial **	55'229	71%	5%	5%	10%	2%	7%
Tortorano et al. [8]	1997-1999	ECMM	Europe	2089	52%	7%	13%	13%	2%	13%

### Antifungal susceptibility

Rates of reduced antifungal susceptibility or resistance ranging from < 5% to > 30% have been reported. The antifungal susceptibility of 2,085 *Candida *isolates to echinocandins (anidulafungin, micafungin) to new azoles (posaconazole, voriconazole) and to fluconazole were tested in the SENTRY survey according to the new Clinical and Laboratory Standard Institute (CLSI) breakpoints [10]. In *C. albicans*, no resistance to the five antifungals was observed. In contrast, resistance rates for *C. glabrata *were reported to be: fluconazole 5.6%, posaconazole 3.7%, voriconazole 3.5%, anidulafungin 2.4%, and micafungin 1.9%, respectively. *C. parapsilosis *was found to be resistant to fluconazole in 5% of the isolates. *C. tropicalis *was resistant to fluconazole in 3.2% of isolates, to posaconazole in 0.9%, and to voriconazole in 2.9%. Finally, *C. krusei *was resistant in 2.5% of cases to voriconazole, whereas no resistance to posaconazole and to the two echinocandins was found. In Denmark, the proportion of fully susceptible species decreased from 79.7% to 68.9% [9]. Leroy et al. reported a decreased susceptibility to fluconazole in 17% of isolates from 180 French ICUs [7].

### Selective pressure of antifungals on species distribution

Preexposure to antifungals, such as prophylaxis, in particular with azoles, and to a lesser extent with echinocandins, has been associated with the occurrence of breakthrough infections with resistant *Candida *species. Whereas *C. glabrata *and *C. krusei *have been classically observed in these settings, other resistant non-*albicans Candida *species are being increasingly observed [17,18]. This was recently confirmed in a large prospective multicenter study conducted by the French Mycosis Study Group in 2,441 candidemic patients reporting that preexposure to fluconazole (159 episodes) or caspofungin (61 episodes) was associated with a higher proportion of less drug-susceptible *C. glabrata *or *C. krusei *(odds ratio (OR), 2.45; 95% confidence interval (CI), 1.39-4.31) and *C. parapsilosis*, *C. glabrata*, or *C. krusei *(OR, 4.79; 95% CI, 2.47-9.28), respectively [19]. These observations are not only of epidemiological interest, but the decreased *in vitro *antifungal susceptibility has been showed to be associated with increased morbidity and mortality in both immunocompromised and critically ill patients [7,20]. Monitoring of resistance plays an important role for updating treatment recommendations designed to improve patients' outcome.

### Impact of invasive candidiasis

Candidemia typically occurs in colonized patients who accumulate other risk factors, such as major surgery, intravascular catheters, and antibacterial exposure, during a prolonged ICU stay [1,21]. It occurs at a median of 22 days after hospital admission compared with 13 days for *Escherichia coli *and 16 days for *Staphylococcus aureus *bacteremias according to the U.S. population-based SCOPE study [5]. It occurred 14 (interquartile range, (IQR), 5-25) days and 19 (± 3) days after ICU admission, in a survey of a university hospital from Paris and in the EPIC II study, respectively [14,22].

Candidemia is associated with significant morbidity, which is reflected by a long ICU and hospital stay, ranging between one and several weeks [7,14]. The overall mortality in patients with invasive *Candida *infections is high: 42.6% in the EPIC II study [22]; 35.2% at 12 weeks in the PATH study [12]; 37.9% in the ECMM study [8]; and 53.4% in non-ICU vs. 85.9% in ICU patients in the Brazilian SCOPE study [11]. In the PATH study, the highest mortality has been reported in *C. krusei *infections (52.9%) and the lowest in *C. parapsilosis *infections (23.7%), whereas intermediate rates were reported for *C. albicans *(35.6%), *C. glabrata *(38.1%), and *C. tropicalis *(41.1%) [12]. Similar differences were found in the ECMM and the French surveys [7,8]. Significant differences in mortality in age groups also were reported: 16.8% in patients 0-19 years of age, 31.3% in 19-65 years of age, and 52.7% in > 65 years of age [12]. Mortality higher than 80% was reported in candidemic patients with septic shock [23]. The mortality attributable to candidemia ranged from 5-49% according to the different types of studies (retrospective vs. prospective), patients (ICU vs. non-ICU, age), and healthcare settings [8,24,25].

A substantial difference in mortality between patients who receive appropriate antifungal therapy (< 5%) and those without appropriate therapy (25-40%) was observed in patients with septic shock [23]. Therapy of candidemia delayed beyond 12 h after sampling of blood has been associated with an increase of in-hospital mortality from < 20% to 40% [26,27]. Because incubation accounts for the majority of the time elapsed between sampling of blood cultures and starting antifungal therapy, these data highlight the need for new noninvasive tools for anticipating diagnosis of invasive candidiasis in high-risk patients, which may play a key role for early and targeted empirical or preemptive treatment strategies [28-30].

### Pathogenesis of invasive candidiasis

**Figure 1:**
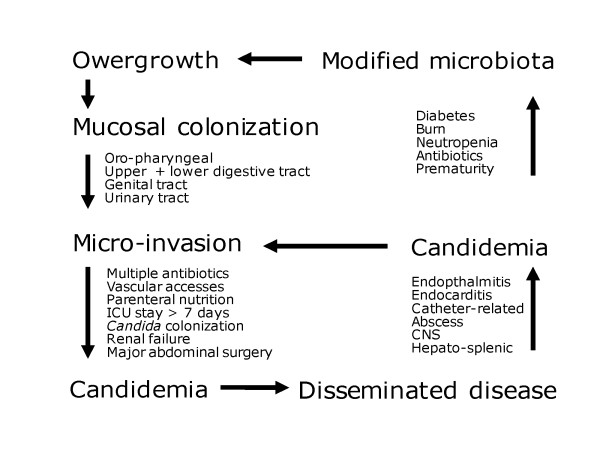
**Pathophysiology of invasive candidiasis**.

During past decades, many risk factors associated with the development of invasive candidiasis have been identified (Table 2) [21,31-33]. Among them, *Candida *colonization plays a key role in the pathogenesis of invasive candidiasis. Selective pressure trough antibacterial therapy alters the microbiota, resulting in overgrowth of *Candida *species on skin and mucosal surfaces [1]. Invasive procedures that disrupt natural skin or mucosal barriers, such as intravascular catheters, gastrointestinal tract surgery, and chemotherapy-associated mucositis, as well as decreased host defenses, in particular neutropenia, facilitate local invasion and further candidemia (Figure 1).

**Table 2:** Risk factors associated with the development of invasive candidiasis

Colonization of several body sites
Broad-spectrum antibiotics
Immunosuppression
Neutropenia
Burns (> 50%)
Disruption of physiological barriers in the digestive tract
Major abdominal surgery
Surgery of the urinary tract in presence of candiduria
Major trauma (ISS > 20)
Parenteral nutrition
Hemodialysis
APACHE score II > 20
Central venous catheter
Candiduria > 10^5 ^cfu/ml
Young and old ages
Diabetes
Renal failure
Recent surgery
Urinary catheter
Vascular catheters
Prolonged ICU stay (> 7 days)
Multiple transfusions

Host defenses against colonization of mucous membranes by *Candida *and invasion of tissues and/or dissemination via the bloodstream rely on distinct immunological mechanisms [34]. Recognition of fungi-associated molecular patterns involves several classes of pattern-recognition receptors. Toll-like receptors (TLRs) 2 and 4 recognize fungal cell-wall structures (mannans) and induce the production of proinflammatory cytokines [35]. Beta-1,3 glucans are sensed by dectin-1, a member of the C-type lectin family of receptors. Activation of the signal transduction pathways downstream of these receptors ultimately lead to the production of a complex array of mediators, including proinflammatory cytokines (such as TNF and IL-1) involved in adaptive immune response [36]. CD4+ T cells have been shown to play a critical role in host defenses against Candida infections. The interferon (IFN)-γ-mediated Th1 response stimulates the production of specific anti-*Candida *immunoglobulins, whose role for prevention and clearance of infection remains to be elucidated [37]. These recent findings open new perspectives for identifying subgroups of patients at higher risk of developing invasive candidiasis and who may benefit from more specifically targeted preventive or preemptive antifungal strategies and/or of immunomodulating approaches [36].

### Diagnosis of candidiasis in critically ill patients

Only 5-15% of patients are colonized by *Candida *spp. at ICU admission, but this proportion progressively increases with time to 50-80% as a result of prolonged exposure to many risk factors, such as major surgery, parenteral nutrition, dialysis, and antibiotics [33,38-40]. However, only 5-30% of colonized patients will develop invasive candidiasis, which is usually a late-onset ICU acquired infection [7,14]. As the clinical differentiation between colonized and infected critically ill patients remains difficult to assess, the utility and cost-effectiveness of colonization surveillance cultures remain unclear [41-43].

Two main types of *Candida *infections predominate. Candidemia occurs generally after several days or weeks of ICU stay, whereas *Candida *peritonitis more closely follows a complicated abdominal surgery. Conventional blood cultures, as well as cultures of other sterile body sites, albeit late and insensitive, remain the key diagnostic tools to identify *Candida *to the species level, and allow to test the activity of various antifungal agents.

Cultures from sites other than blood or normally sterile body fluids are nonspecific and reflect colonization in the majority of cases. Blood cultures become positive in a minority of patients with deep-seated candidiasis and often only late in the course of infection [7,14,22]. Conventional identification of *Candida *to the species level usually requires 1 to 3 days after detection of fungal growth in blood cultures. The recent development of new laboratory techniques (fluorescent *in situ *hybridization [FISH], and matrix-assisted laser desorption ionization time of flight mass-spectrometry [MALDITOF-MS]), significantly help to reduce the delay to species level identification, and thus allow an earlier choice of appropriate antifungal therapy [44].

Invasive candidiasis other than candidemia is difficult to diagnose. Clinical signs suggestive of invasive candidiasis did not differ from those of other nosocomial infections. More specific manifestations, such as retinal emboli (cotton whole) or hepatosplenic lesions are rare or only observed in cancer patients after neutrophils recovery [45,46]. Tissue sampling often requires invasive procedures at high risk of complications and has a low diagnostic yield, especially in patients who have received empirical therapy.

### Nonculture-based methods

The delay between ICU admission and the occurrence of deep-seated *Candida *infections allows both to identify patients at increased risk and to attempt to detect early onset of infections. Several biomarkers are currently tested with this strategy, either based on antigen-antibody detection or on fungal DNA detection in serum or blood.

Commercially available antigen-based test target a *Candida *specific cell-wall component, mannan, or a nonspecific fungal element, beta-D-glucan. Both have a moderate sensitivity (60% for mannan, 83% when combined to anti-mannan antibodies, 65-80% for beta-D-glucan) and are intended to be used as a screening strategy two to three times per week. Mostly tested in oncohematology patients, their value in an ICU population is still insufficiently documented [47,48].

The second noncultural approach relies in detecting the presence of *Candida *DNA in the blood of at-risk patients. The major hurdle to this technique is the lack of commercial easy-to-use methods and the relative low sensitivity of this approach, due to several factors (low quantity of *Candida *cells in the blood, inhibitors due to blood cells). An additional difficulty is the "gold standard" generally used in evaluations, blood cultures, which itself lacks sensitivity. Available comparative studies in ICU patients are limited, showing a sensitivity equal to/or slightly lower than blood cultures (75-100% compared with blood cultures) [49,50].

### Clinical prediction of invasive candidiasis in critically ill patients

Despite continuous progress and developments in this field, the absence of laboratory-based method currently available at the bedside has imposed pragmatic clinical approaches based on the appreciation of the dynamics of colonization and/or of the combination of less specific risk factors [51].

### Colonization-based assessment of the risk of invasive candidiasis

Documentation of increasing amounts of *Candida *spp. in semiquantitative cultures from multiple sites has been found to predict the subsequent development of invasive candidiasis [21,52,53]. It has been suggested that the presence of *Candida *spp. in more than two body sites may justify the start of antifungal therapy [54,55]. However, critically ill patients are being colonized progressively during their ICU stay. The accuracy of a single-point assessment is low and such rule may be responsible for overuse of antifungals [56]. As initially proposed by Pittet et al. and confirmed by other investigators, a periodic evaluation of the dynamics of colonization in surgical patients at risk may predict more accurately the development of an invasive candidiasis [21,57-60].

In a prospective cohort study of surgical critically-ill patients, Pittet et al. assessed the degree of colonization by measuring daily the colonization index defined as the ratio of the number of distinct body sites colonized with genotypically identical strains of *Candida *over the total number of sites tested [21]. Eleven of 29 heavily colonized patients developed invasive candidiasis. The severity of the underlying conditions and the degree of colonization independently predicted the occurrence of invasive candidiasis. The average *Candida *colonization index was 0.47 for colonized vs. 0.7 for infected patients, respectively (*p *< 0.01). Furthermore, a threshold ≥0.5 identified all infected patients at an average of 6 days before the diagnosis of invasive candidiasis.

The usefulness of the colonization index has never been demonstrated in a large prospective clinical trial, but its potential clinical value has been suggested in at least nine studies. Dubau et al. reported that an invasive candidiasis developed in only 1 of 35 surgical patients in whom empirical antifungals were prescribed when the index reached 0.5 and that it decreased rapidly in the 34 other patients [61]. Garbino et al. prospectively observed a decrease of the index in a group of critically ill patients receiving antifungal prophylaxis [42]. In contrast, it increased with time in those who received a placebo. Differences reached statistical significance between the two groups after 7 days. Chabasse et al. found a correlation between quantitative urine cultures above 10^4 ^cfu/mL and a colonization index ≥0.5 [62]. Charles et al. reported significantly higher values in medical patients (0.74 ± 0.31) compared with surgical patients 0.45 ± 0.4 (*p *= 0.01) [57]. The index increased significantly by 0.1 during the ICU stay (*p *= 0.016) and the threshold of 0.5 was reached in 36 (39.1%) of 92 nonsurgical ICU patients staying > 7 days; 6 of them developed invasive candidiasis [63]. Hematological malignancy, duration of exposure to broad-spectrum antibiotics, fungal colonization at entry, and candiduria predicted an increase in the colonization index. In contrast, the duration of exposure to antifungals was significantly associated with its decrease. Compared with an historical cohort of 455 controls, the rate of invasive candidiasis decreased from 7% to 3.6% in a cohort of 478 surgical ICU patients who received preemptive antifungal treatment if the corrected colonization index was > 0.4 [64]. This strategy avoided the development of ICU-acquired invasive candidiasis. Normand et al. reported a significant reduction of the colonization index in a cohort of 98 patients mechanically ventilated > 48 hours randomized to receive prophylaxis by oral nystatin [65]. Agvald-Öhman et al. showed that increases of colonization index after major abdominal surgery were significantly correlated with the development of an IC [59]. Senn et al. reported a significant decrease of the colonization index in critically ill patients empirically treated with caspofungin after recurrent gastrointestinal perforation/anastomotic leakage or acute necrotizing pancreatitis [60].

Although these observations strongly suggests that the colonization index may be used to identify among colonized critically ill patients those who are susceptible to benefit from early initiation of antifungal therapy, this strategy is work-intensive, expensive, and difficult to implement on a routine basis at the bedside [66]. Its cost-effectiveness and usefulness for the management of critically ill patients remains to be proved in prospective comparative clinical trials [30]. In addition, limited data are available for nonsurgical patients.

### Risk of invasive candidiasis assessed by scores or predictive rules

Scoring systems or predictive rules that combine clinical risk factors and information for *Candida *colonization have been recently proposed [67-69]. A risk-based "Candida score" has been developed by Leon et al. in a prospective cohort of 1,699 ICU patients staying more than 7 days [68]. Surgery (OR, 2.71; 95% CI, 1.45-5.06), multifocal colonization (OR, 3.04; 95% CI, 1.45-6.39), total parenteral nutrition (OR, 2.48; 95% CI, 1.16-5.31), and severe sepsis (OR, 7.68; 95% CI, 4.14-14.22) significantly predicted invasive candidiasis. By attributing one point of each risk factor, the score for a cutoff value of 2.5 had a sensitivity and specificity of 81% and 74%, respectively. The usefulness of this risk-factor based "Candida score" has been demonstrated further to rule out invasive candidiasis [70]. In a multicenter cohort of 1,007 patients staying for more than 7 days, only 13 of 565 (2.3%) patients with a score < 3 points developed a candidiasis, corresponding to a negative predictive value of 98%. In this series, a linear progression of the risk of invasive candidiasis and higher score was further observed. The accuracy of a colonization index ≥0.5 (relative risk, 5.98, 95% CI, 3.28-10.92) was lower than a Candida score ≥3 (relative risk, 5.98; 95% CI, 3.28-10.92).

Using a similar conceptual approach, Paphitou et al. identified retrospectively individual risk factors for the development of invasive candidiasis in a cohort of 327 surgical ICU patients to build a predictive rule [71]. The rate of invasive candidiasis was 17% for patients staying more than 3 days in ICU, with the combination of diabetes mellitus, dialysis, total parenteral nutrition, and exposure to broad-spectrum antibiotics compared with 5% for those lacking these characteristics (*p *< 0.01). Fifty-two percent of patients met this rule, which captured 78% of those who developed invasive candidiasis. For patients staying 4 days or more, Ostrosky-Zeichner et al. refined this preliminary clinical prediction rule in a large multicenter retrospective study [72]. Any systemic antibiotics or the presence of a central venous catheter during the 3 preceding days and at least two of the preceding risk factors was able to identify patients with a risk of invasive candidiasis of at least 10%. However, with a sensitivity of 34% this rule captured only one third of cases of invasive candidiasis. The usefulness of a risk-factors-based predictive rule has been suggested in a medical ICU where antifungals were empirically prescribed accordingly [73]. Thirty-six (2.6%) of all patients admitted received antifungals empirically, allowing a significant decrease of the rate of fungal catheter-related bloodstream infections from 3.4 to 0.79 episodes per 1,000 catheter-days. The sensitivity of such clinical predictive rule was markedly improved (66%) with a maintained specificity (87%) by taking into account the presence of *Candida *colonization at time of its assessment [74]. This new rule is currently investigated in a randomized, placebo-controlled, pilot study on empirical therapy with caspofungin in high-risk ICU patients (MSG-04 in mixed patients, INTENSE study in surgical patients, http://www.clinicaltrials.gov).

However, the common characteristics of risk scores and clinical rules is not their relatively low positive predictive value for diagnosing invasive candidiasis but their high negative predictive value for ruling out infection. This may allow withholding a number of unnecessary antifungal treatments in critically ill patients.

### Management of invasive candidiasis in critically ill patients

**Figure 2:**
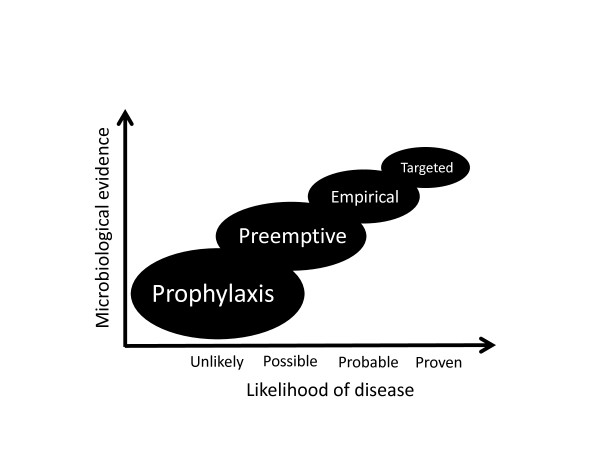
**Concept of antifungal treatments in critically ill patients**.

Rapid initiation of appropriate antifungal therapy has been shown to reduce mortality in patients with candidemia [24,75]. Prophylaxis should strongly be restricted to very specific subgroups of patients in whom it has been demonstrated to be useful (Table 3). Preemptive therapy for colonized patients or those with high-risk scores and empirical therapy in septic patients not responding to appropriate antibacterial treatment are possible early interventions (Figure 2) [28,60].

**Table 3:** Criteria used for antifungal prophylaxis in adult critically ill patients

Study	Criteria used for prophylaxis	Antifungal used for prophylaxis	Invasive candidiasis	Commentary
**Positive prophylactic studies**				
*Slotman et al. 1987 [77]	Abdominal surgery + ≥ 3 risk factors	Ketoconazole 200 mg/d PO Placebo	0/27 (0%) 5/30 (17%)^†^	Costs: $4,800 vs. $10,000^†^ LOS: 6.0 vs. 12.5 days^†^
*Savino et al. 1992 [78]	Surgical patients + hypermetabolism	Nystatin/norfloxacin PO Placebo	6/25 (24%) 13/21 (62%)^‡^	NI per patient: 0.9 vs. 2.0^†^
Desai et al. 1992 [79]	Severely burned patients	Nystatin/polymyxin SDD No prophylaxis	34/1042 (3.3%) 0/1439 (0%)^†^	Superficial infections: 59 (21%) vs. 22 (10%)^†^
Eggimann et al. 1999 [39] *	Abdominal surgery + tertiary peritonitis	Fluconazole 400 mg/d IV Placebo	1/23 (9%) 7/20 (35%)^‡^	Candida peritonitis 1 (4%) vs. 7 (35%)^†^
Pelz et al. 2001 [43] *	Surgical patients + LOS > 3 days	Fluconazole 400 mg/d PO Placebo	11/130 (8%) 20/130 (15%)^†^	> 75% colonized at randomization
Garbino et al. 2002 [42] *	Mechanically ventilated > 96 h	Fluconazole 100 mg PO + SDD Placebo + SDD	4/103 (4%) 10/101 (10%)^‡^	Candidemia: 9 vs. 1 (RR 0.1; CI 0.02-0.74)^†^
Jacobs et al. 2003 [80] *	ICU + septic shock	Fluconazole 200 mg IV/d Placebo	0/32 (0%) 1/39 (3%)^‡^	Mortality significantly reduced in peritonitis
He et al. 2003 [81]	Severe acute pancreatitis	Fluconazole 100 mg IV/d Placebo	2/22 (9%)^†^ 7/23 (30%)^†^	Mortality 2/2 (100%) Mortality 3/7 (43%)
**Negative prophylactic studies**				
Savino et al. 1994 [78]	Surgical patients + LOS > 2 days	Nystatin 2 × 10^6 ^4 ×/d PO Ketoconazole 200 mg/d PO Clotrimazole 10 mg 3 ×/d PO No prophylaxis	5/75 (7%) 1/65 (2%)^‡^ 1/80 (1%)^‡^ 2/72 (3%)^‡^	
Ables et al. 2000 [82]*	Surgical patients + LOS > 2 days + other risk factors	Fluconazole 3 mg/kg 3 ×/w Placebo	8/60 (13%) 11/59 (19%)^‡^	
Sandven et al. 2002 [40]*	Surgery for peritonitis	Fluconazole 400 mg/d IV Placebo	- -	Mortality rates NS 4/53 (8%) vs. 8/56 (14%)
Schuster et al. 2008 [83]*	ICU ≥ 4 d + Fever > 4 d under broad-spectrum antibiotics + APACHE II ≥ 16	Fluconazole 400 mg/d IV Placebo	6/122 (5%) 11/127 (9%)^‡^	

*Prospective randomized double-blind

^†^p < 0.05

^‡^Not significant

### Practical approach to early diagnosis of invasive candidiasis in critically ill patients

**Figure 3:**
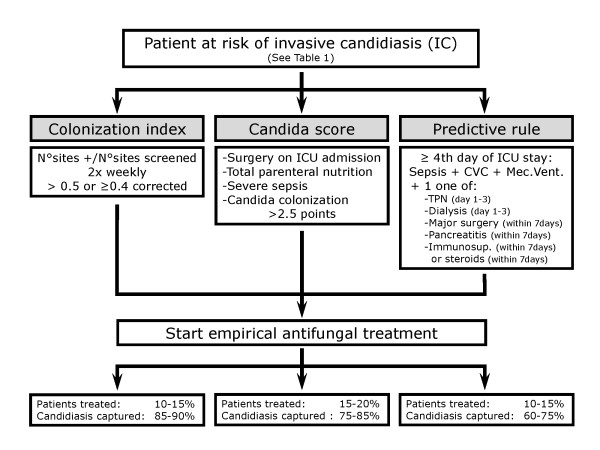
**Practical approach of patient at risk of invasive candidiasis**. Suggested algorithm to be applied in patients at risk of invasive candidiasis after having check that they are among those susceptible to benefit from prophylaxis (see Table 3) or evaluated to be at a risk level too low to justify antifungal prophylaxis, such as early after extended abdominal surgery or secondary peritonitis.

Although recognized as a strong risk factor, colonization, which may occur early after ICU admission, does not justify the start of empirical antifungal treatment [76]. Despite promising preliminary results, biomarkers are currently only available for research purpose. Accordingly, clinicians should continue to combine risk factors and the dynamic of colonization to try to identify early critically ill patients susceptible to benefit from early empirical antifungal treatment (Figure 3).

## Competing interests

Pertinent to this article, PE received research grants and/or educational grants and/or speaker's honoraria and/or consultant's honoraria's from the (in alphabetic order): Astellas, Merck, Sharp & Dohme-Chibret, and Pfizer. JB has no disclosures regarding this manuscript. OM received unrestricted research grants and/or educational grants and/or speaker's honoraria and/or consultant's honoraria from (in alphabetical order): Foundation for the Advancement in Medical Microbiology and Infectious Diseases FAMMID, Associates of Cape Code, BioMérieux-Cepheid, Bio-Rad, Essex Schering-Plough, Gilead, Merck, Sharp & Dohme-Chibret, Novartis, Pfizer, Roche Diagnostics, Wako.

## Authors' contributions

PE, JB and OM designed the structure of this review, wrote dedicated original sections and contributed to finalize MS.
